# Interfacial Microstructure in W/2024Al Composite and Inhibition of W-Al Direct Reaction by CeO_2_ Doping: Formation and Crystallization of Al-Ce-Cu-W Amorphous Layers

**DOI:** 10.3390/ma12071117

**Published:** 2019-04-04

**Authors:** Zheng Lv, Changhui Mao, Jian Wang, Qiushi Liang, Shuwang Ma, Zhimin Yang, Jian Yang, Yang Li

**Affiliations:** Advanced Electronic Materials Institute, GRIMAT Engineering Institute Co., Ltd., No.11 Xingkedongda Street., Beijing 101407, China; lvzheng1988@126.com (Z.L.); wangjian@grinm.com (J.W.); liangqiushi@grinm.com (Q.L.); mashuwang@grinm.com (S.M.); power@grinm.com (Z.Y.); yangj@grinm.com (J.Y.); liyang@grinm.com (Y.L.)

**Keywords:** W/2024Al composite, interfacial reaction, W-Al compounds, CeO_2_ doping, Al-Ce-Cu-W amorphous

## Abstract

In this work, interfacial microstructure in W/2024Al composite and inhibition of the W-Al direct reaction by CeO_2_ doping were investigated. The composites were prepared through powder sintering, and after preparation the composites were treated by annealing at 823 K. For the prepared W/2024Al composite, a multi-phase thin layer composed of WAl_12_ and WAl_5_ compounds were formed at the interface due to the W-Al direct reaction. While doping CeO_2_ in the composite, Al-Ce-Cu-W amorphous substituting of W-Al compounds were formed at the interfacial reaction layer. In an annealed state, the composite with CeO_2_ doping shows a significant inhibitory effect on W-Al compounds, which was attributed to the crystallized layer that evolved from Al-Ce-Cu-W amorphous as an interfacial obstacle. The crystallization product for Al-Ce-Cu-W amorphous layer was identified as bcc-structure Al-Ce-Cu-W phase without any binary/ternary Ce-containing phases. Therefore, by doping CeO_2_ in W/2024Al composite, W-Al direct reaction was markedly inhibited during both preparation and annealing.

## 1. Introduction

The interfacial microstructure has played effective and important roles in the exploration of particle reinforced aluminum matrix composites (PRAMC) and improvement of their thermal stability, mechanical and physical properties. In recent decades, a number of researchers have attempted to optimize the interfacial structure of PRAMC used for many applications such as multifunctional electronic packaging [1], thermal management [2], transport industry [3,4], aerospace industry [5,6], and so on. W/2024Al composite, which could be used for radiation shielding with a relative lower density, has attracted more attention in the interfacial microstructure because of its possible reactions at the W/Al interface during preparation and annealing. Most of the W-Al intermetallic compounds (e.g., WAl_12_, WAl_5_) are brittle and will deteriorate the mechanical properties of composites as interfacial reaction products. Moreover, the formation of W-Al interfacial compounds could lead to the obvious volume expansion of composites, i.e., poor thermal stability. In previous research, hot-pressed W/2024Al and WC/2024Al composites were prepared at different temperatures [7]. The research has shown that the WC substituting of W has led to a marked increase of W-Al reaction onset temperature which was attributed to the formation of interfacial Al_4_C_3_ layers. The Al_4_C_3_ layers inhibited the W-Al reactions as physical obstacle at interfaces. In principle, W-Al direct reaction was undesirable within the W/2024Al composite.

Up to date, there have been several research studies devoted to the microstructures and properties of PRAMC with doping of rare earth oxides [8,9,10,11,12]. However, the question, does doping of rare earth oxides have an influence on interfacial reactions has not been discussed in detail in this research. In this work, W/2024Al composite and the counterpart with CeO_2_ doping were prepared by powder sintering, and after preparation the composites were treated by annealing. Subsequently, the microstructure at the pure W/Al interface and the effect of CeO_2_ doping on the interfacial microstructure were investigated in greater detail. Here we have assumed that CeO_2_ doping in the W/2024Al composite could result in the formation of new phases at the W/Al interfaces and thus the W-Al direct reaction can be inhibited to a certain extent. 

This paper aims to provide detailed microstructures at the interfaces in the W/2024Al composite, and attempts to inhibit direct W-Al reaction through CeO_2_ doping. For characterizations, we investigated the phase compositions (by XRD), thermal stability (by DSC) and interfacial microstructures (by TEM) of composites in both the prepared and annealed states.

## 2. Materials and Methods

### 2.1. Preparation of Composites

In this work, W/2024Al composite and its counterpart with CeO_2_ doping (denoted as ‘AW’ and ‘AWC’) were prepared by powder sintering. The sintering process contains three sub-processes, i.e., preparation of powder mixtures, vacuum degassing (VD) and hot isostatic pressing (HIP).

Atomized 2024Al powders, tungsten powders (~5 μm, Zhuzhou Cemented Carbide Group Co., Ltd, Zhuzhou, China) and CeO_2_ powders (~5 μm, Yixing Xinwei Co., Ltd, Yixing, China) were used as starting materials. The composition of 2024 Al powders is provided in Table 1. Prior to VD, 2024Al-W powder mixture with 40 wt.% W and 2024Al-W-CeO_2_ powder mixture with 40 wt.% W and 3 wt.% CeO_2_ were prepared. The SUS 304L tapered cylinder and balls were selected as the blending medium for powder mixtures. Blending parameters of mixtures were selected as follows: Ball to powder ratio 1:1, rotation speed 20 rpm, total blending time 24 h, under an air atmosphere.

For encapsulation, the above two powder mixtures were cold isostatic pressed at 150 MPa and then filled into two pure Al containers with 1 mm thickness. Then the containers were sealed with a plug by welding and degassed through the ventilation tube on the plug at 723 K until the vacuum degree reached 10^−3^ Pa. After VD, the samples were placed in a hot isostatic press and heated to 723 K at a heating rate of 5 K/min. A maximum hydrostatic pressure of 100 MPa was applied on the samples with a holding time of 2 hours. After preparation, AW and AWC were annealed according to the following procedure: 5 K/min until 823 K, 5 h holding under vacuum, furnace cooling to room temperature.

### 2.2. Characterizations

To evaluate the structure of composites in prepared and annealed states, X-ray diffraction (XRD, PANalytical B.V., Almelo, Netherlands) was employed. The instrument X’PERT-PRO MPD with Cu *Kα* radiation was used. For a determination of the W-Al reaction onset temperature during continuous heating, two prepared composites was studied by differential thermal analysis (NETZSCH DSC 404F3, NETZSCH-Gerätebau GmbH, Selb, Germany) and thermal expansion analysis (NETZSCH DIL 402PC, NETZSCH-Gerätebau GmbH, Selb, Germany). The samples were heated to 950 K with heating rate of 5 K/min under a high purity argon gas flow. Interfacial microstructures were examined and analyzed carefully by High Resolution Transmission Electron Microscope (FEI Tecnai G2 F20, FEI Company, Hillsboro, OR, USA) equipped with an energy dispersive spectroscopy (EDS, FEI Company, Hillsboro, OR, USA). Selected area electron diffraction (SAED, FEI Company, Hillsboro, OR, USA) was utilized to identify the crystalline structures of interfacial phases. Prior to TEM investigations, the thin foils were prepared by an ion beam milling technique.

## 3. Results and Discussion

### 3.1. XRD Patterns and Thermal Measurements

**Figure 1:**
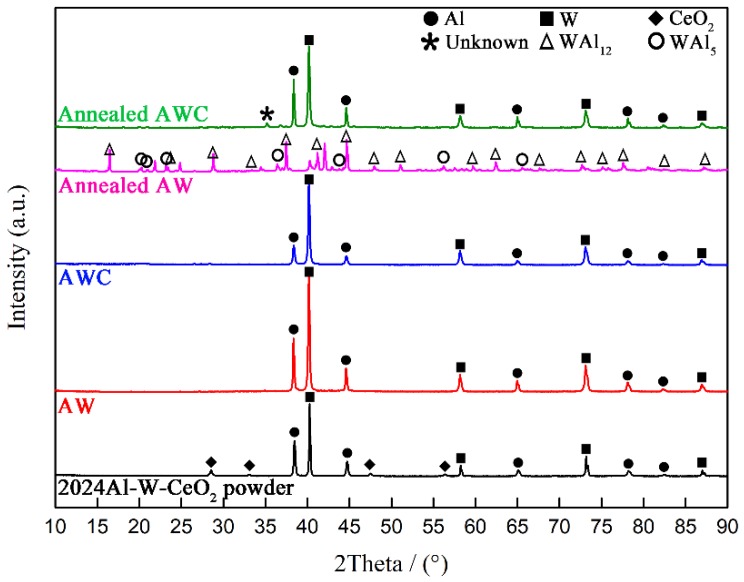
XRD patterns of 2024Al-W-CeO_2_ powder mixture, two prepared composites and their annealed counterparts.

Figure 1 shows the XRD patterns of 2024Al-W-CeO_2_ powder mixture, two prepared composites (AW and AWC) and two annealed counterparts, respectively. For the powder mixture, characteristic diffraction peaks of three starting materials were clearly presented in the diffraction pattern. For two prepared composites, there were only diffraction peaks of Al and W phases, that is, no obvious W-Al reactions occurred during preparation. By comparing the two prepared composites, it was apparent that the characteristic peaks of Al were sharply decreased in AWC. Adding further to the puzzle was the fact that the CeO_2_ phase was undetectable in the pattern of prepared AWC. From the above analyses, we speculated that one or more interfacial phases consisted predominantly of Al with subordinate Ce were formed in the AWC during preparation.

After annealing at 823 K, there were intensive interfacial reactions in AW which is indicated by diffraction peaks of WAl_12_ and WAl_5_ compounds. For annealed AWC, however, no diffraction peaks of W-Al compounds were found, while a trace of an unknown phase appeared as the reaction product. Certainly, CeO_2_ doping inhibited the W-Al direct reactions during annealing. The inhibition of W-Al reactions during annealing might be relevant to the interfacial phases formed in the prepared state.

**Figure 2:**
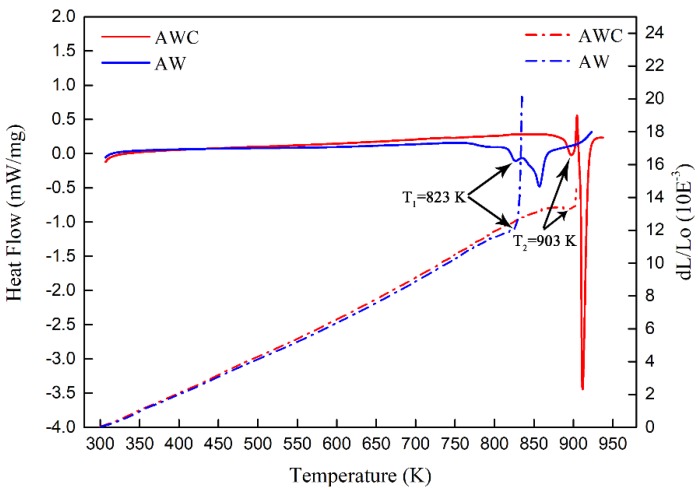
DSC traces and thermal expansion curves of AW and AWC. DSC traces are represented with solid lines and thermal expansion curves are represented with dash lines.

In order to determine the reaction onset temperature during continuous heating, DSC traces and thermal expansion curves of AW and AWC were recorded. Figure 2 shows the DSC traces and thermal expansion curves of AW and AWC heated to 950 K with a heating rate of 5 K/min. The DSC traces are represented with solid lines and the thermal expansion curves are represented with dash lines. Exothermic peaks indicated that two obvious reactions occurred during the DSC testing for both AW and AWC. The reaction onset temperatures of AW and AWC (at the middle of the first exothermic peak) are denoted as T_1_ and T_2_, respectively. It can be clearly seen that the reaction onset temperature was pushed up from 823 K to 903 K due to CeO_2_ doping. In addition, the obvious reaction was confirmed as the direct consequence of the sharp expansion for both composites.

### 3.2. TEM Investigations at Interfaces

#### 3.2.1. Interfacial Microstructure in Prepared AW

**Figure 3:**
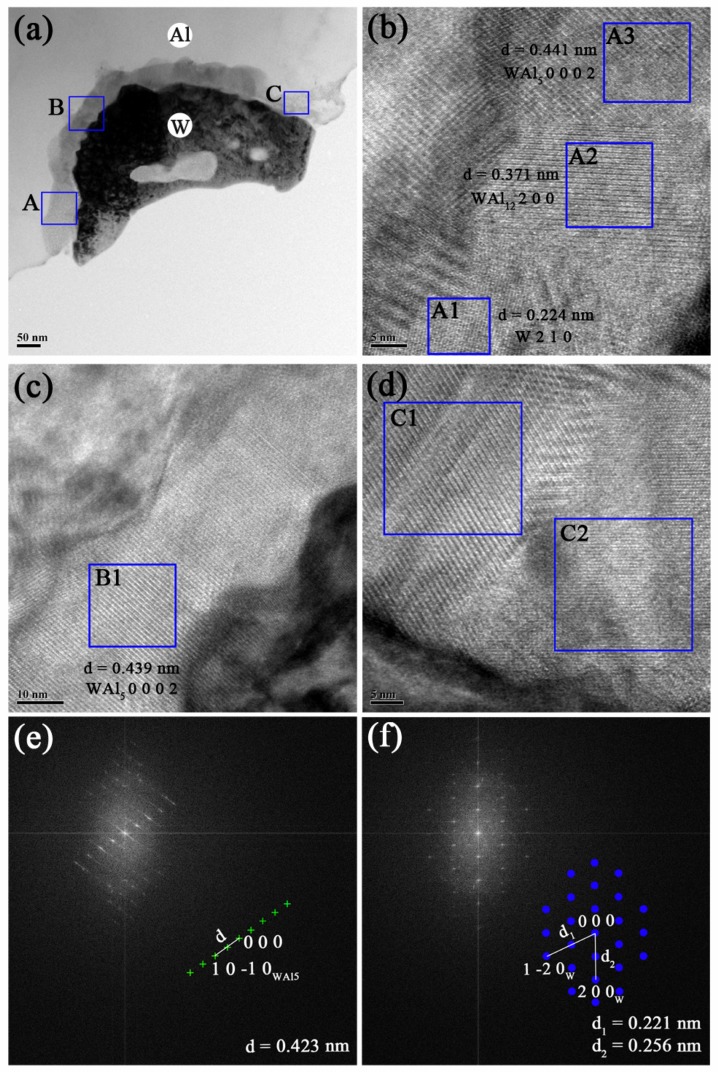
(**a**) TEM morphology of W/Al interface in prepared AW, and three areas ‘A’, ‘B’, ‘C’ are marked in (**a**). (**b**) high-magnification of area ‘A’ with sub-areas labelled with ‘A1’, ‘A2’, ‘A3’. (**c**) high-magnification of area ‘B’ with sub-area labelled with ‘B1’. (**d**) high-magnification of area ‘C’ with sub-areas labelled with ‘C1’, ‘C2’. FFT graphs of ‘C1’ and ‘C2’ are shown as (**e**) and (**f**), respectively.

For a definite study of pure W/Al interface, it is important to have information on the interfacial microstructure in AW. Figure 3 shows the detailed analyses of TEM microstructures at the pure W/Al interface. Even though no apparent W-Al compounds were detected by XRD analysis in the prepared AW (Figure 1), a thin reaction layer with 50–60 nm thickness can be observed at the pure W/Al interface as shown in Figure 3a. Within the reaction layer, three typical areas labelled with ‘A’, ‘B’, ‘C’ were selected to be further magnified as shown in Figure 3b, Figure 3c and Figure 3d, respectively.

In area ‘A’ (Figure 3b), three sub-areas are labelled with ‘A1’, ‘A2’ and ‘A3’. The crystalline interplanar distances in these three sub-areas were calculated to be 0.224 nm (W _(2 1 0)_, PDF#47-1319), 0.371 nm (WAl_12 (2 0 0)_, PDF#08-0331) and 0.441 nm (WAl_5 (0 0 0 2)_, PDF#30-0046), respectively. C. Mao found that the interfacial diffusion in W-Al couple was controlled by the diffusion of W into Al during hot-pressing of WC/2024Al, and the formation of WAl_5_ at 720 °C was attributed to following transformation [7]:

$$5WAl_{12}+7W\;\overset{720\;{}^{\circ}C}{\to }12WAl_{5}$$

Thus, the appearance of W within the reaction layer indicated that the diffusion of W toward the Al matrix during preparation. In area ‘B’ (Figure 3c), lattice fringe of WAl_5 (0 0 0 2)_ was observed with a d value (d = 0.439 nm) calculated in the sub-area ‘B1’. In area ‘C’ (Figure 3d), two sub-areas are labelled with ‘C1’ and ‘C2’. Identification of the phases in these two sub-areas were conducted on the fast Fourier transform (FFT) analysis. The calculated d value (d = 0.423 nm) in FFT of C1 (Figure 3e) corresponded to WAl_5 (1 0 -1 0)_, and the calculated two d values (d_1_ = 0.221 nm, d_2_ = 0.256 nm) in FFT of C2 (Figure 3f) corresponded to W _(1 -2 0)_ and W _(2 0 0)_, respectively. By comparing ‘B1’ and ‘C1’, this indicated that there was a discontinuous formation of WAl_5_ phase at the continuous W/Al interface.

#### 3.2.2. Interfacial Microstructure in the Prepared AWC

**Figure 4:**
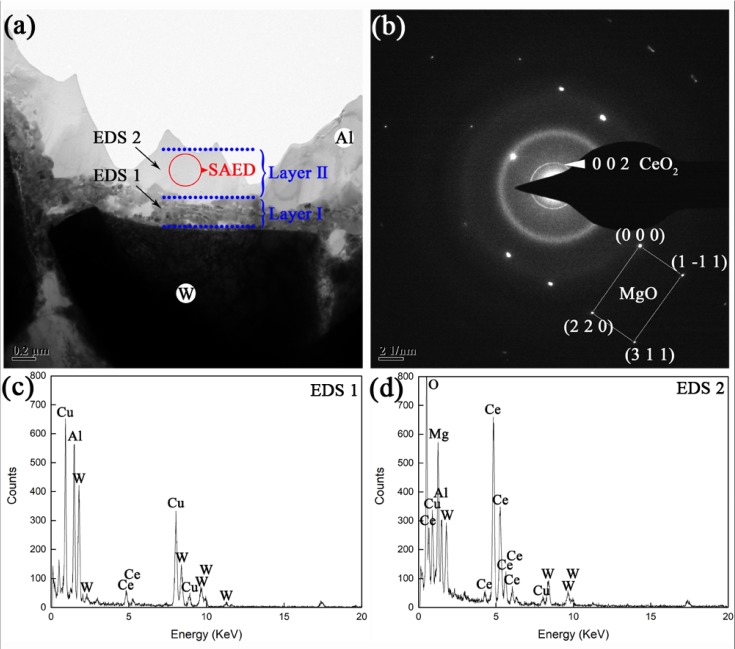
(**a**) TEM micrograph of W/Al interface in prepared AWC, and two EDS points ‘EDS1’, ‘EDS2’ are marked in (**a**). (**b**) SAED pattern of the circular area marked in (**a**). EDS analyses are shown as (**c**) and (**d**), respectively.

In order to trace the impact of CeO_2_ doping on microstructure at the W/Al interface, prepared AWC was also examined by TEM. Figure 4 shows the TEM morphology, SAED pattern and EDS analyses of the interface in prepared AWC. The TEM micrograph (Figure 4a) shows a two-layer interfacial structure between W and Al, i.e., a reaction layer near W and a transition layer near Al (denoted as Layer I and Layer II, respectively). The SAED pattern of the circular area labelled in Layer II is shown as Figure 4b.

**Figure 5:**
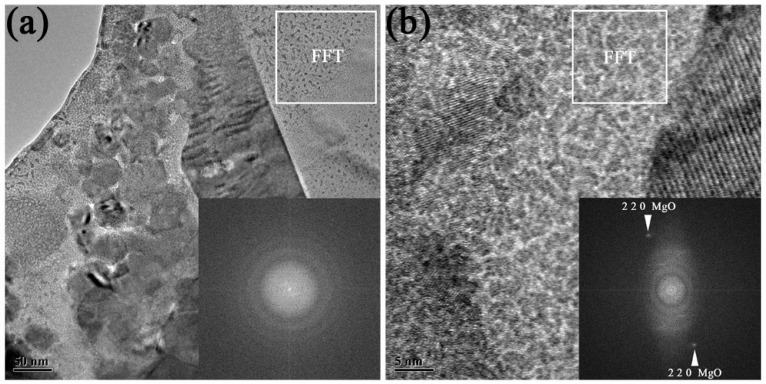
(**a**) and (**b**) show TEM morphologies of W/Al interfaces in prepared AWC. The insets show FFT graphs of marked squares.

**Figure 6:**
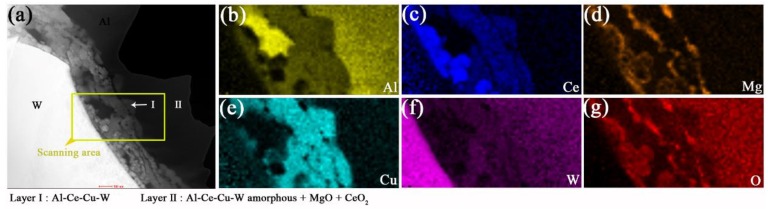
HADDF-STEM-EDS scanning area was marked in (**a**) STEM micrograph. The EDS mapping analyses of six elements ((**b**) Al, (**c**) Ce, (**d**) Mg, (**e**) Cu, (**f**) W, (**g**) O) at the W/Al interface of prepared AWC were displayed.

EDS analyses shows that Layer I and Layer II were composed of Al-Ce-Cu-W (Figure 4c) and Al-Ce-Cu-W-Mg-O (Figure 4d), respectively. From the SAED pattern of Layer II, a broad diffuse ring in the background originated from the electrons scattered in amorphous, while a sharp diffraction ring and a set of diffraction spots corresponded to the electrons scattered from the crystalline lattice of crystallites. The broad diffuse ring indicates an amorphous matrix of Layer Ⅱ. Structural identification of the crystalline diffraction ring and spots yielded a good match with CeO_2_ and MgO, respectively. Thus, Layer II consists of an amorphous matrix with subordinate CeO_2_ and MgO crystallites. As also shown in Figure 5, the FFT analyses of marked squares provided more evidence of the formation of amorphous at interfaces. Figure 6 shows HADDF-STEM-EDS mapping analyses of the six elements (Al, Ce, Cu, W, Mg, O) at the Interface. In the mapping area, the two-layer interfacial structure ‘I–II’ between W and Al is clearly displayed. The EDS analyses demonstrated a composition of Al-Ce-Cu-W for pure amorphous layer ‘I’. Combined with XRD analyses (Figure 1), the interfacial Al-Ce-Cu-W was confirmed as one Al-based amorphous. The same distribution of Mg and O directly proved the formation of the MgO phase. Thus, by doping CeO_2_, Al-Ce-Cu-W amorphous substituting of W-Al compounds were formed as an interfacial reaction layer during preparation.

So far, Al-Ce-TM (TM: Transition metals) amorphous have been widely studied on the basis of liquid-solid amorphization [13,14,15,16,17,18,19,20]. Understandably, it is difficult for W to participate in liquid-solid amorphization due to its ultra-high melting point. However, W was proved to participate in the formation of Al-Ce-TM amorphous through solid-state amorphization. The Al-Ce-Cu-W discovered in this work indicated that the Al-Ce-TM amorphous can also be formed through solid-state amorphization. Certainly, the detailed mechanism involved in Al-Ce-Cu-W amorphization needs further investigation in the future.

#### 3.2.3. Interfacial Microstructure in Annealed AWC

**Figure 7:**
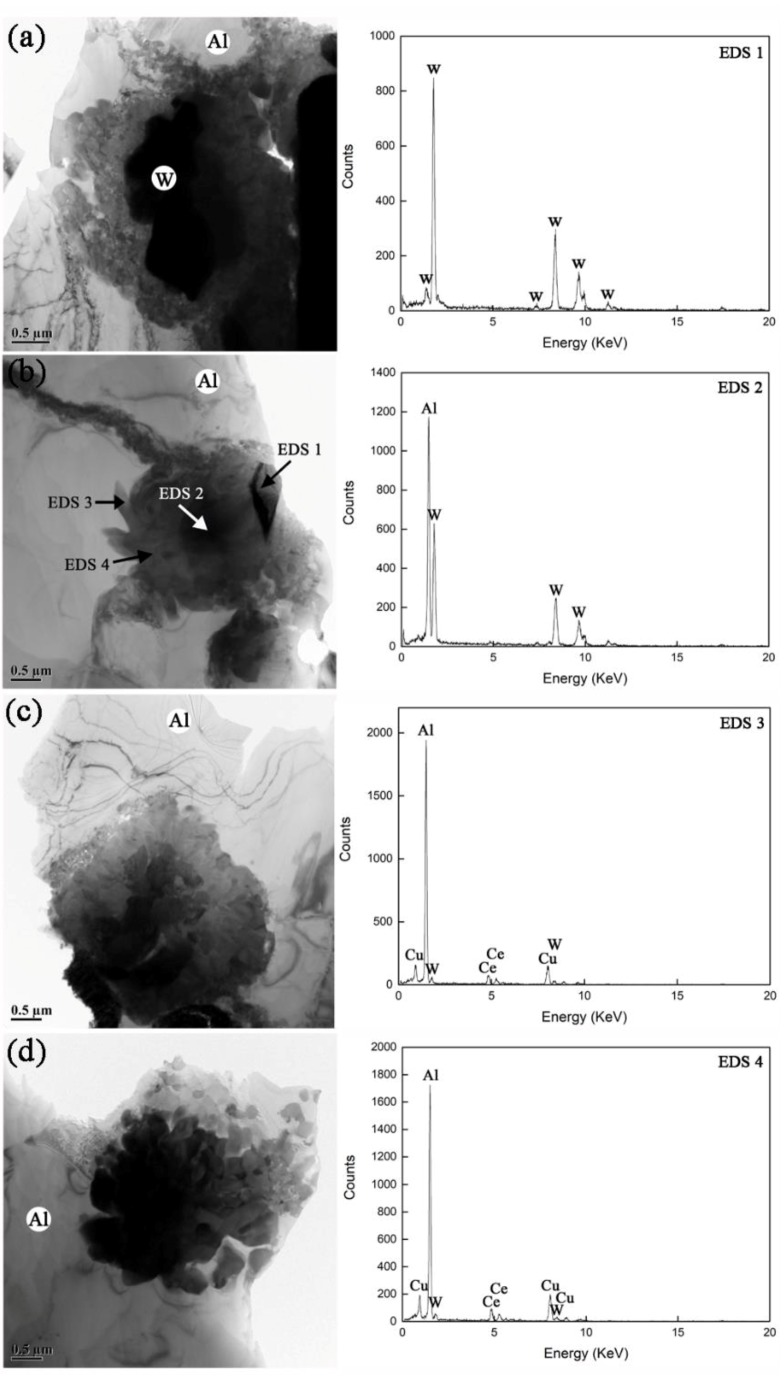
TEM morphologies of interfacial structures in annealed AWC. By comparing with the prepared AWC, the change in interfacial morphology increased from (**a**) to (**b**) and then to (**c**) and finally to (**d**). EDS analyses are displayed at the bottom.

The analyses of XRD patterns and DSC traces revealed that W-Al direct reactions were markedly inhibited by CeO_2_ doping during annealing. For a determination of the inhibition mechanism, it is important to have detailed information on the interfacial microstructure of annealed AWC. With TEM examinations, we investigated the interfaces with representative morphologies as shown in Figure 7. By comparing Figure 4 and Figure 7, it can be seen that the annealed AWC exhibited distinctly different interfacial morphologies with its prepared counterpart. Thus, we speculated that in some cases the former interfacial amorphous was transformed into new phases during annealing.

Evidently, the change in interfacial morphology increased from Figure 7a,b and then to Figure 7c and finally to Figure 7d. Figure 7a shows the interfacial morphology that represented the majority of interfaces, and in which the W particle is relatively stable. Figure 7b shows a three-layer ‘core-shell’ structure at its interface, and EDS points labelled with ‘EDS 1’, ‘EDS 2’ ‘EDS 3’ and ‘EDS 4’ are marked in ‘core’, ‘middle layer’ and ‘shell’, respectively. From the EDS analyses, the elemental compositions of ‘core’, ‘middle layer’ and ‘shell’ corresponded to W, Al-W and Al-Ce-Cu-W, respectively. By comparison with Figure 7a, Figure 7b shows an atrophied W ‘core’ which resulted from the reasonable consumption in the formation of Al-W ‘middle layer’. For ‘shell’, the elemental composition was inherited from the former Al-Ce-Cu-W amorphous in the prepared AWC. In Figure 7c, the W ‘core’ had completely run out and the Al-W ‘middle layer’ was in growth, which led to the structural evolution into a two-layer ‘core-shell’. With the growth of the Al-W ‘core’, Al-Ce-Cu-W ‘shell’ was fragmented into countless ‘petal-shaped grains’ as shown in Figure 7d.

**Figure 8:**
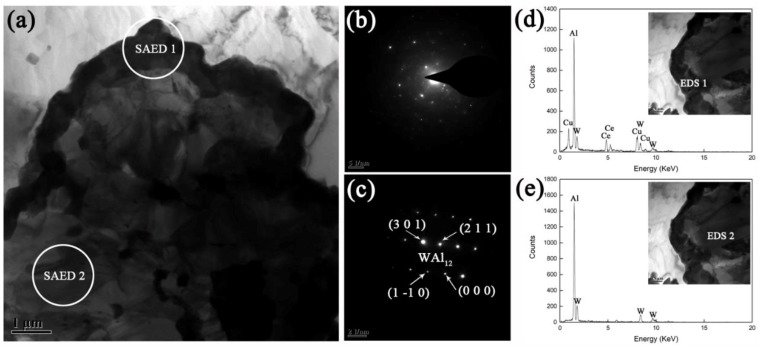
(**a**) TEM morphology of ‘core-shell’ structure in the annealed AWC. (**b**) and (**c**) shows the SAED patterns of circular areas labelled with ‘SAED 1’ and ‘SAED 2’ in (**a**), respectively. EDS analyses of ‘shell’ and ‘core’ are shown as (**d**) and (**e**), respectively.

For microstructural investigation of the two-layer ‘core-shell’, a high-magnification micrograph was selected to be further characterized. Figure 8 shows the TEM micrograph, SAED patterns and EDS analyses of the two-layer ‘core-shell’. EDS analyses of ‘shell’ and ‘core’ (labelled with EDS 1 and EDS 2) shows the should-be compositions of Al-Ce-Cu-W and Al-W, respectively. The SAED pattern involving a part of ‘shell’ (SAED 1, Figure 8b) did not show a broad diffuse ring originating from the electrons scattered in the amorphous. Thus, the former Al-Ce-Cu-W amorphous layer at the interface was crystallized during annealing. It can be clearly seen that the crystalline ‘shell’ is continuous and dense. The SAED pattern involving a part of ‘core’ (SAED 2, Figure 8c) shows a set of diffraction spots that yields a good match with the WAl_12_ phase.

**Figure 9:**
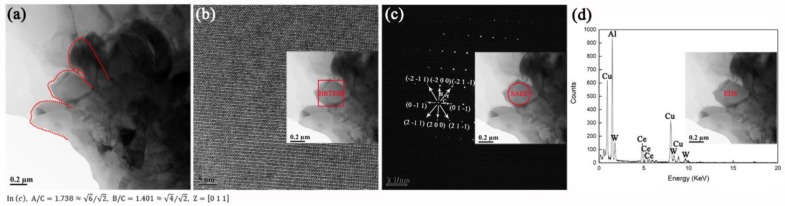
(**a**) TEM morphology of ‘petal-shaped grains’ in annealed AWC. For one single ‘grain’, (**b**) HRTEM micrograph, (**c**) SAED pattern and (**d**) EDS analysis were displayed.

**Figure 10:**
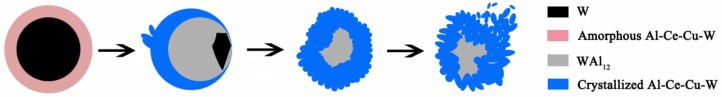
Schematic diagram for the evolution of interfacial structure in AWC during annealing.

In order to further characterize the crystalline ‘shell’, the fragmented ‘petal-shaped grains’ (structural units of ‘shell’) were examined by TEM as shown in Figure 9. In Figure 9a, ‘petal-shaped grains’ are compactly arranged. From the HRTEM micrograph (Figure 9b), the SAED pattern (Figure 9c) and EDS analysis (Figure 9d), the ‘petal-shaped grains’ were confirmed as bcc-structure single crystals with elemental composition of Al-Ce-Cu-W, i.e., the elemental composition of crystalline ‘petal-shaped grains’ was consistent with that of its amorphous predecessor. Thus, the crystallization product for Al-Ce-Cu-W amorphous was identified as bcc-structure Al-Ce-Cu-W phase without any binary/ternary Ce-containing phases such as Ce-Al and Ce-Al-TM. However, such a crystallization behavior was rarely observed in the reported Al-Ce-TM amorphous system [21,22,23,24,25,26,27,28,29,30,31,32,33,34,35,36,37,38,39,40]. The reported crystallization products for Al-Ce-TM amorphous system are summarized in Table 2, and the corresponding crystallization conditions are also listed. From the above research, the crystallization products for the Al-Ce-TM system can be categorized into four types: Fcc-Al phase, Ce-Al phases, Al-TM phases and Ce-Al-TM phases. Generally according to the order in which the products precipitated during annealing, the fcc-Al phase precipitated in the early stage of crystallization and the other decomposition products precipitated in the later stages of crystallization. By contrast, no binary/ternary Ce-containing phases were decomposed from the Al-Ce-Cu-W amorphous during annealing in this work. The schematic diagram for the evolution of the interfacial structure during annealing is illustrated in Figure 10. Thus during annealing, the inhibitory effect of CeO_2_ doping on W-Al compounds was attributed to crystallized Al-Ce-Cu-W layer as an interfacial obstacle.

## 4. Conclusions

In this work, the interfacial microstructure in W/2024Al composite and inhibition of W-Al direct reaction by CeO_2_ doping were investigated. The composites were prepared through powder sintering, and after preparation the composites were treated by annealing at 823 K. For the prepared W/2024Al composite, a multi-phase thin layer composed of WAl_12_ and WAl_5_ compounds were formed at the interface due to W-Al direct reaction. By doping CeO_2_ in the composite, Al-Ce-Cu-W amorphous substituting of W-Al compounds were formed as an interfacial reaction layer. In the annealed state, the composite with CeO_2_ doping shows a significant inhibitory effect on W-Al compounds, which was attributed to the crystallized layer that evolved from the Al-Ce-Cu-W amorphous as an interfacial obstacle. The crystallization product for Al-Ce-Cu-W amorphous was identified as bcc-structure Al-Ce-Cu-W phase without any binary/ternary Ce-containing phases. As a summary, the W-Al direct reaction within the W/2024Al composite was markedly inhibited through CeO_2_ doping during both preparation and annealing.

## Figures and Tables

**Table 1:** Chemical analysis (wt.%) of 2024 aluminum alloy powders used in this work.

Element	Cu	Mg	Mn	Fe	Si	Al
Chemical composition	4.20	1.48	0.58	0.16	0.087	Balance

**Table 2:** Crystallization products of reported Al-Ce-TM (TM: Transition metals) amorphous system [21,22,23,24,26,27,28,30,32,33,35,36,38,39,40]. The corresponding crystallization conditions are also listed.

Al-Ce-TM Amorphous System	Crystallization Products	Corresponding Crystallization Conditions
Al_90_Fe_5_Ce_5_ [21]	Fcc-Al	Pre-existing in amorphous quenched from 1173 °C
Al_88_Ni_9_Ce_2_Fe_1_ [22]	Fcc-Al	Annealing at 138 °C under 1.07 GPa in pressure vessel
	Fcc-Al + Al_3_(Ni, Fe) + Al_11_Ce_3_	Annealing at 306 °C under 0.85 GPa in pressure vessel
Al_84.2_Ni_10_La_2.1_Ce_2.8_Pr_0.3_Nd_0.6_ [23]	Fcc-Al + Al_3_Ni	Continuous heating to 550 K in DSC instrument
	Fcc-Al + Al_3_Ni + Al_11_(La, Ce)_3_	Continuous heating to 615/680 K in DSC instrument
Al_87.5_Ni_7_Mm_5_Fe_0.5_ [24]	Fcc-Al	Annealing at 433 K
	Fcc-Al + Al_11_(La, Ce)_3_	Annealing at 593 K
	Fcc-Al + Al_11_(La, Ce)_3_ + Al_3_Ni + Al_4_Ce	Annealing at 613 K
Al_87_Co_10_Ce_3_ [26]	Fcc-Al + Co_2_Al_9_	Continuous heating to 262 °C in DSC instrument
	Fcc-Al + Co_2_Al_9_ + Al_4_Ce	Continuous heating to 274/303/342 °C in DSC instrument
Al_82_Fe_5_Ni_5_Ce_8_ [27] (powders)	Al_11_Ce_3_ + Al_3_(Fe, Ni)	Annealing at 673 K for 10 h
Al_87_Ni_6_Ce_7_ [28]	Fcc-Al + unknown metastable phase 2	Isochronal heating to 563 K in DSC instrument
Al_85_Ni_6_Ce_9_ [28]	Unknown metastable phase 3	Isochronal heating to 570 K in DSC instrument
Al_85_Ni_10_Ce_5_ [30]	Fcc-Al + Al_3_Ni + Al_4_Ce	Annealing at ~727 K for 1 h
[Al_85_Ni_10_Ce_5_]_95_Ag_5_ [30]	Fcc-Al + Al_3_Ni + Ag_3_Al_17_Ce_5_	Annealing at ~723 K for 1 h
[Al_85_Ni_10_Ce_5_]_95_Pd_5_ [30]	Fcc-Al + Al_3_Ni + unidentified phases	Annealing at ~861 K for 1 h
Al_86_Ni_6_Y_6_Ce_2_ [32]	Fcc-Al	Isochronal annealing up to 548 K in DSC instrument
	Fcc-Al + Al_3_Ni	Isochronal annealing up to 618 K in DSC instrument
	Fcc-Al + Al_3_Ni + Al_11_Re_3_	Isochronal annealing up to 688 K in DSC instrument
Al_86_Ni_9_(La_1−x_Ce_x_)_5_ [33] (x = 0−1)	Fcc-Al	Annealing to the end of first DSC exothermic peak
Al_85.5_Ni_9.5_(La_1-x_Ce_x_)_5_ [33] (x = 0.2−1)	Fcc-Al + Al_4_NiCe + Al_11_Ce_3_	Annealing to the end of first DSC exothermic peak
Al_86_Ni_10_MM_4_ [35,36,40]	Fcc-Al	Annealing up to 553 K in DSC instrument
	Fcc-Al + Al_11_MM_3_ + Al_3_Ni	Annealing up to 623/714 K in DSC instrument
Al_88_Ni_10_MM_2_ [36,40]	Fcc-Al	Annealing up to 553 K in DSC instrument
	Fcc-Al + Al_11_MM_3_ + Al_3_Ni	Annealing up to 714 K in DSC instrument
Al_90_Fe_5_Ce_5_ [38]	Fcc-Al + icosahedral phases	Annealing at 613 K for 2 h
(Al_90_Fe_5_Ce_5_)_100−x_Ti_x_ [38] (x = 4, 8)	Fcc-Al + icosahedral phases	Annealing at 613 K for 2 h
(Al_90_Fe_5_Ce_5_)_88_Ti_12_ [38]	Fcc-Al + icosahedral phases + Al_20_CeTi_2_	Pre-existing in amorphous
Al_86_Ni_10_Zr_2_MM_2_ [39]	Fcc-Al	Annealing up to ~570 K in DSC instrument
	Fcc-Al + Al_11_MM_3_ + Al_3_Ni + Al_3_Zr	Annealing up to ~690 K in DSC instrument
Al_86_Ni_9.5_Cu_0.5_RE_4_ [40]	Fcc-Al + Al_11_MM_3_ + Al_3_Ni	Full-crystallization annealing in DSC instrument
Al_86_Ni_9_Cu_1_RE_4_ [40]	Fcc-Al + Al_11_MM_3_ + Al_3_Ni	Full-crystallization annealing in DSC instrument
Al_86_Ni_7.5_Cu_2.5_RE_4_ [40]	Fcc-Al + Al_11_MM_3_ + Al_3_Ni + Al_3_MMCu	Full-crystallization annealing in DSC instrument

In Reference [24], Mm contains 55 wt.% Ce, 25 wt.% La, 10 wt.% Nd, 7 wt.% Pr and 3 wt.% Fe. In References [35,36,40], Mm/RE contains 55 wt.% Ce, 25 wt.% La, 15 wt.% Nd and 5 wt.% Pr. In Reference [39], MM contains 49 wt.% Ce, 26 wt.% La, 20 wt.% Nd and 5 wt.% Pr.
